# Supplementary material for: Measurement of the combined quantum and electrochemical capacitance of a carbon nanotube
Source: Nat Commun. 2019 Aug 9;10:3598. doi: 10.1038/s41467-019-11589-9 (PMC6689019; doi:10.1038/s41467-019-11589-9)
Supplement: Supplementary file 1 — Supporting Information [file 41467_2019_11589_MOESM1_ESM.pdf]

Measurement of the combined quantum and electrochemical capacitance of a carbon nanotube

Jinfeng Li et al.

Supplementary Figures:

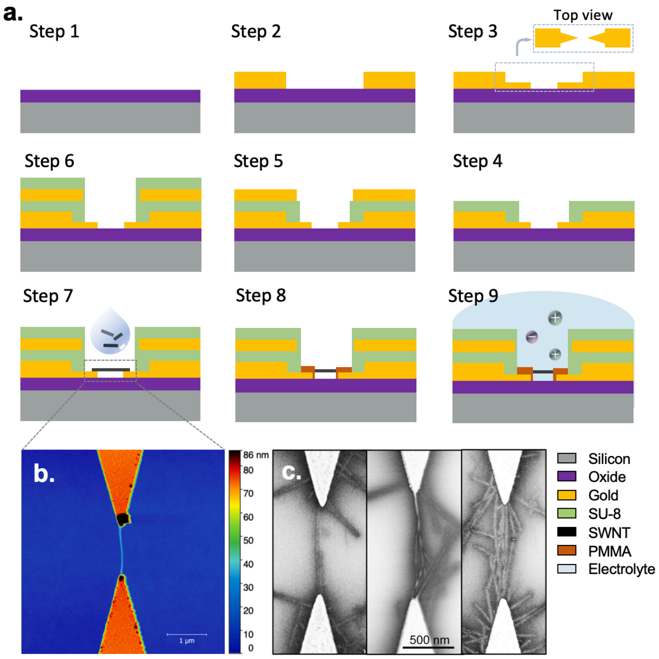

Supplementary Figure 1: Device fabrication. (a) Fabrication process for a SWNT FET device with the integrated shield. Step 1: A highly doped silicon wafer with a 300 nm oxide layer. Step 2: Source-drain electrodes fabrication using standard photolithography. Step 3: Fine-tip electrodes fabrication using e-beam lithography. Step 4: Dielectric layer fabrication. Step 5: Top shielding layer fabrication. Step 6: Top passivation layer fabrication. Step 7: Nanotube deposition using dielectrophoresis (DEP). Step 8: Final PMMA passivation layer with only the nanotube exposed. Step 9: Electrolyte solution brought in contact with the nanotube acting as the top-liquid gate. (b, c) Topographic images of the SWNTs imaged by AFM and SEM. The SWNTs were DEP-attached to the source-drain electrodes. Multiple amounts of SWNTs (1~100 nanotubes) between the source and drain electrodes were observed.

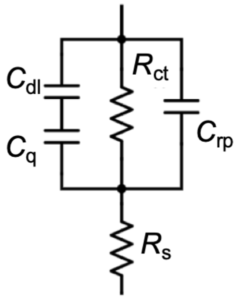

Supplementary Figure 2: Detailed circuit between a nanotube and an electrolyte solution.

Supplementary Note 1: Details of C_{meas}.

The measured capacitance C_{meas} contains the quantum capacitance C_q and the double layer capacitance C_{dl} in series as the main components (Supplementary Figure 2). In parallel with the capacitances, there is the charge transfer resistance R_{ct} which is much larger compared to the capacitive impedance in our measurement window and can be ignored. Also, in parallel, there is the remaining parasitic capacitance C_{rp} at non-shielded space, which is constant and can be simply subtracted as a background. Finally, the electrode contact resistance and the resistance of the electrolyte is much smaller than that of the tube-liquid interface, hence it can be ignored. Note that the double layer capacitor is not a perfect capacitor; it has a phase shift value different from −90°, which can be phenomenally described as a constant phase element (CPE).[1–3] The phase shift phenomenon was also observed in liquid-gated graphene studies.[4] The ratio between the imaginary part and the real part of the measured current after the background subtracting suggests that the phase shift of the current is at the value ~ 70°.

Supplementary References:

1. Brug, G. J., van den Eeden, A. L. G., Sluyters-Rehbach, M. & Sluyters, J. H. The analysis of electrode impedances complicated by the presence of a constant phase element. *J. Electroanal. Chem.* **176**, 275–295 (1984).
2. Pajkossy, T. Impedance of rough capacitive electrodes. *J. Electroanal. Chem.* **364**, 111–125 (1994).
3. Bisquert, J., Garcia-Belmonte, G., Bueno, P., Longo, E. & Bulhões, L. O. S. Impedance of constant phase element (CPE)-blocked diffusion in film electrodes. *J. Electroanal. Chem.* **452**, 229–234 (1998).
4. Crosser, M. S., Brown, M. A., McEuen, P. L. & Minot, E. D. Determination of the thermal noise limit of graphene biotransistors. *Nano Lett.* **15**, 5404–5407 (2015).
